# A combination of drug, behavioral and surgical therapy to relieve from severe obesity complicated with multiple organs failure: A case report

**DOI:** 10.1097/MD.0000000000041846

**Published:** 2025-03-28

**Authors:** Feng Feng, Jiaqi Zhang, Yuqi Gao, Qiaoni Ye, Guiqi Wang, Jingfeng Gu, Liping Peng

**Affiliations:** a Ministry of Education Key Laboratory of Molecular and Cellular Biology, Hebei Collaborative Innovation Center for Eco-Environment, College of Life Sciences, Hebei Normal University, Shijiazhuang, Hebei, China; b Department of Gastrointestinal Surgery, The First Hospital of Hebei Medical University, Shijiazhuang, Hebei, China.

**Keywords:** bariatric surgery, case report, combined treatment, complication, severe obesity

## Abstract

**Rationale:**

The prevalence of obesity and its associated complications is on the rise globally. Bariatric surgery has proven to be a very effective method to induce sustained weight loss as well as reduce obesity-related diseases, such as type 2 diabetes, hypertension and hyperlipemia.

**Patient concerns:**

The 30-year-old female patient, with BMI at 79.2 kg/m^2^ and abdominal circumference at 199 cm, gained weight continually, felt chest tightness and shortness of breath for 10 days.

**Diagnoses:**

The patient was diagnosed with severe obesity complicated with multiple organs failure.

**Interventions:**

The patient was given a personal diet plan to lose weight. Besides, she was given noninvasive ventilator to improve ventilation dysfunction during night sleep, and hypoglycemic therapy and treatment of pulmonary hypertension were given to improve heart failure. When the weight was effectively reduced, bariatric surgery was performed to reduce weight more remarkable.

**Outcomes:**

One month after surgery, the overall weight of the patient was significantly reduced with meliorative BMI at 58.6, and the blood glucose was significantly reduced to the normal level and her overall physique was thin down and slimmer than before.

**Lessons:**

Multi-disciplinary combination and comprehensive treatment can effectively reduce the risk of surgery. Moreover, surgery should not be performed as soon as possible for patients with extremely severe obesity, and preoperative weight loss can improve the organs function of patients.

## 1. Introduction

Obesity is a metabolic disease caused by genetic and environmental factors. It is defined that a body mass index of 25.0 to 29.9 is considered overweight, and BMI reaching to 40 or higher is considered severe obesity.^[1]^ The prevalence of obesity has increased significantly around the world over the last 2 decades. Approximately 39 million children under the age of 5 years old had overweight or obesity in 2020.^[2]^ It was estimated that 57.8% of the world population would be overweight or obese by the year 2030 if the current trends continue, and the prevalence of obesity would reach to 18% among men and 21% among women by 2025.^[3]^ Obesity causes various diseases and has the potential to cause complications. For instance, most of obese people suffer from endocrine disorders, which could lead to excessive production of melanin, and the accumulation of melanin on the skin surface causes acanthosis nigricans.^[4]^ What’s more serious, obesity brings about many complications, such as type 2 diabetes, cardiovascular and cerebrovascular disease, kidney disease, sleep apnea, cancer, even shortens life expectancy.^[5]^

To cure severe obesity, the surgical treatment could be considered primarily. Compared with diet or exercise, the operation methods are simple, low complication-rate, obvious weight-losing result and long-lasting effect.^[6]^ Bariatric surgery improves metabolism through changing gastrointestinal anatomy. Laparoscopic sleeve gastrectomy (LSG), Roux-en-Y gastric bypass and biliopancreatic diversion with duodenal switch are the most widely performed surgeries. LSG could reduce the volume of the stomach and decrease the food intake, which was mainly used for severe obesity case originally.^[7]^ Besides, clinical evidence showed that LSG had satisfying effect on weight loss and metabolism improvement, therefore, LSG is gradually used as first-tier surgery widely.^[8]^

**Figure 1.**
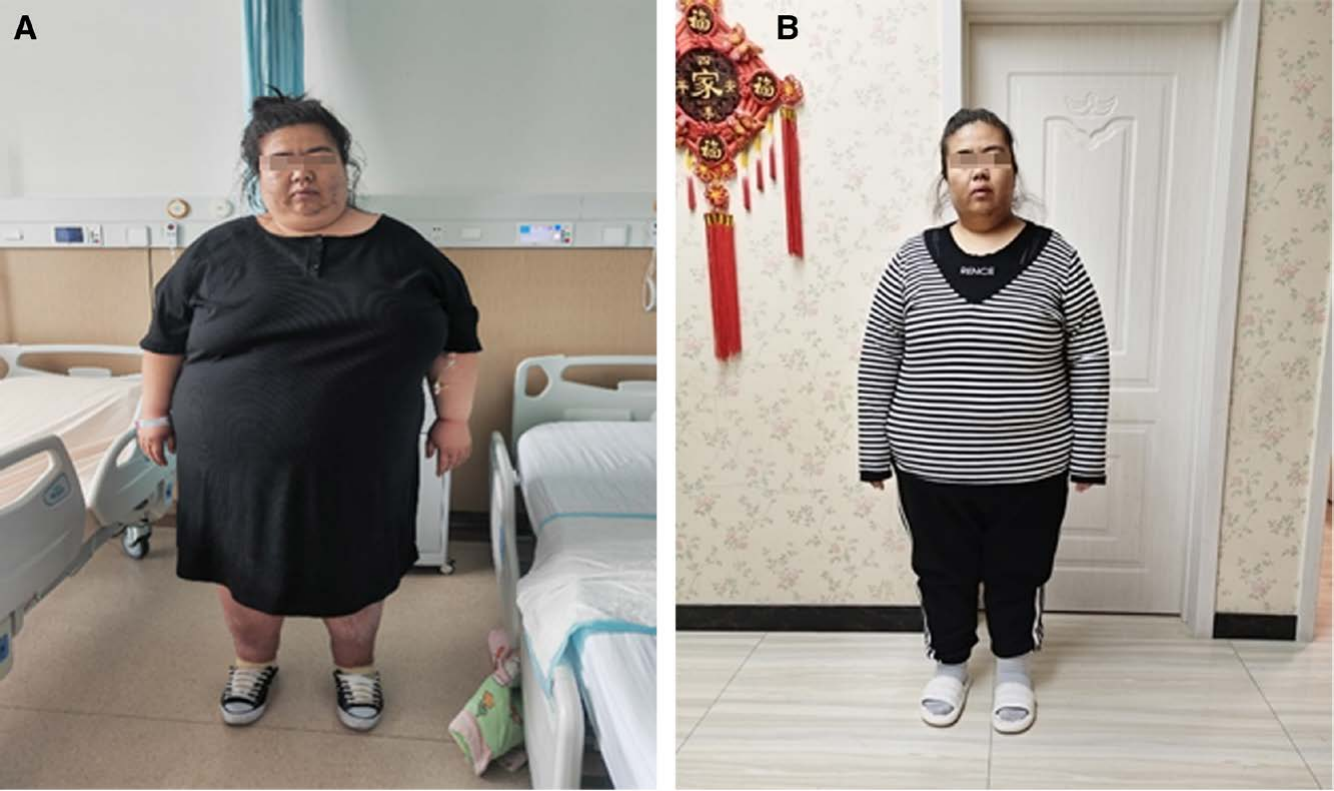
The combination of drug, behavioral and surgical therapy had a satisfying effect on losing weight of patient. (A) Preoperation. (B) Postoperation.

In this study, a young woman with severe obesity complicated with multiple organs failure was cured with combination of drug, behavioral and surgical therapy. After surgery, not only the overall weight was significantly reduced, but also other physiological indexes were improved, such as the number of red blood cells, white blood cells, bacterium and the concentration of bilirubin and microalbuminuria (Table 1). It was exciting to find that the blood glucose was significantly reduced to the normal level and her overall physique was thin down and slimmer than before (Fig. 1).

**Table 1:** Comparison of biochemical indexes.

Parameter	Presurgery	After-surgery	Reference range
Weight (kg)	203	150	/
BMI (kg/m^2^)	79.2	58.6	18.5–24
Abdomen circumference (cm)	199	168	<80
Fasting blood-glucose (mmol/L)	9	6.8–7.5	3.9–6.1
2-h postprandial blood glucose (mmol/L)	13	9.7–10.6	<7.8
COL	Dark yellow	Light yellow	/
RBC (μL)	45	3	0–17
WBC (μL)	159	47	0–28
BACT (μL)	15,350	3745	0–340
HYAL (μL)	21	1	0–1
MUCS (μL)	34	0	0–46
PRO (g/L)	2+	Negative	Negative
BLD (Ery/μL)	2+	Weakly positive	Negative
BIL (μmol/L)	1+	Negative	Negative
LEU (Leu/μL)	Weakly positive	Negative	Negative
NIT	2+	Negative	Negative
SG	1.030	1.020	1.003–1.030
pH	6.0	5.5	4.5–.8.0
MALB (mg/L)	150	10	0–80
CRE (mmol/L)	26.5	4.4	4.4–17.7
A:C (mg/mmol)	3.4–33.9	<3.4	0–3.4
PASP (mm Hg)	73	40	18–25
CA125 (U/mL)	479.40	146.50	0–35.00

A:C = microalbumin/creatinine, BACT = bacterium, BIL = bilirubin, BLD = blood occult, CA125 = carbohydrate antigen 125, CLO = color, CRE = creatinine, HYAL = hyaline casts, LEU = leukocyte esterase, MALB = microalbuminuria, MUCS = mucous strands, NIT = nitrite, PASP = pulmonary arterial systolic pressure, pH = power of hydrogen, PRO = protein, RBC = red blood cell, SG = specific gravity, WBC = white blood cell.

## 2. Material and methods

A 30-year-old woman was presented with progressive weight gain within 2 months, chest distress and short breath for ten days. On pre-hospitalization physical examination, her weight was 203 kg, height was 160 cm and BMI was 79.2 kg/m^2^. Through observation, it was found that her abdominal skin was rough and black, and abdominal circumference was 199 cm. Besides, her abdominal subcutaneous fat was accumulated obviously, and both lower extremities had pitting edema. On laboratory examination, her microalbuminuria level was 150 mg/L, uric acid level was 516.8 μmol/L, pulmonary systolic pressure was 73 mm Hg and CA125 content was 479.40 U/mL (Table 1). In the endocrine examination, both cortisol and ACTH level were normal. Analysis of patient’s body composition demonstrated that the patient was overweight, with the percentage of total body fat in the normal population reached to 98%. She used to take weight-loss pills while the effect was unsatisfying. Besides, she didn’t have the familial inheritance for her parents were normal in weight.

Combined with the above examination results, the patient was diagnosed as follows. Metabolic syndrome, type II respiratory failure, cardiac failure, pulmonary arterial hypertension, respiratory acidosis, type II diabetes, pulmonary heart disease, obesity-related glomerulopathy, hyperuricemia, sleep apnea syndrome. This patient had cardiac failure, respiratory failure, and severe pulmonary hypertension. Her blood oxygen was 85% at quiescent condition so that she would have wheeze and dyspnea when walking <100 m continuously. Her abdomen circumference too overlarge to conduct conventional CT scan, making the preoperative evaluation extremely difficult.

The treatment difficulties faced by patients were as follows: Firstly, the patient had cardiac failure, respiratory failure, severe pulmonary hypertension, respiratory acidosis and extremely low blood oxygen at quiescent condition. If surgical operation performed, the tracheal intubation may not be removed after surgery, and the ventilator may not be removed for a long time, which could lead to high surgical risk. Secondly, the patient’s CA125 was extremely high, but CT examination of the chest, abdomen and pelvis could not be completed to exclude the possibility of internal tumor. Finally, the patient was respiratory failure along with cardiac failure, and would face the risk of survival if she failed to lose weight.

As a result, the treatment methods were as follows: Firstly, according to the patient’s weight, she was given a personal diet plan, such as protein powder, vegetable juice and fruit juice, which could not only meet the daily energy and calories, but also help her lose weight effectively. Secondly, she was given noninvasive ventilator to improve ventilation dysfunction during night sleep, and balloon blowing was practiced to improve cardio-pulmonary function. Hypoglycemic therapy and treatment of pulmonary hypertension were given to improve heart failure. Through preoperative medication and behavioral therapy, the patient successfully lost 38.5 kg of weight in 12 days. The symptoms of wheeze and dyspnea were significantly alleviated, the blood oxygen level at quiescent condition was up to 95%, and the severe pulmonary hypertension was reduced to mild pulmonary hypertension. The abdominal circumference was significantly reduced, which was benefit to CT examination of the chest, abdomen and pelvis. CA125 level was significantly reduced to except digestive system and gynecological tumors. The weight loss of the patient had reached to plateau and could not continue to lose weight in a short time. After multi-disciplinary consultation, surgery was considered possible.

The operation was conducted after 14 days hospitalized. A hernia needle was insert into a 1 cm curved incision at the upper margin of the navel, and CO_2_ gas was injected into it. When pneumoperitoneum pressure reached 12 mm Hg, 10 mm trocar was inserted, laparoscope was implanted, making 5 and 12 mm incisions in the left and right upper quadrant. Make sure that ascites was not found in the abdominal cavity. And check whether bilateral ovaries, uterus, bowel, liver, gallbladder, spleen stomach morphology and texture were normal, whether the gastric serous membrane was smooth. Following the greater curvature of the stomach, an ultrasound scalpel was used to dissociate the greater curvature of stomach from 3 cm above the pylorus to His-angle. Paries posterior gastricus and the whole side of greater curvature of the stomach were both dissociated. 36Fr support tube was inserted by oral, an electric endoscopic linear cutting stapler was placed in. A gastrotomy was performed lengthways along the greater curvature of the stomach about 1 cm from the support pipe and 3 cm above pylorus until fundus of stomach to the bottom of the stomach. Part of gastric tissue in greater curvature of the stomach and support tube were both removed separately. 3-0 absorbable suture was used to suture the lateral margin of the greater curvature of the stomach and performed hemostasis thoroughly. Besides, although the operation was completed successful, it was obvious that the abdominal fat was large and the operating space was narrow, making it more difficult than other obese people.

The operation was successful, which lasted for 65 minutes and the intraoperative blood loss was about 30 mL. In order to survive the perioperative period safely, the patient was transferred to the intensive care unit. The endotracheal tube was removed 4 hours after surgery. On the first day after surgery, blood glucose level of the patient was improved. She didn’t need subcutaneous insulin injection or oral hypoglycemic agents to control blood glucose. On the second day after surgery, she could vent anally, walk down and take care of herself. On the third day after surgery, she started to eat a small amount of protein powder. On the sixth day after surgery, her fasting blood-glucose was 6.80 to 7.50 mmol/L and postprandial blood glucose level was 9.70 to 10.60 mmol/L. Digestive tract radiography showed no clear signs of contrast agent leakage around the stomach. One month after surgery, the patient’s weight was 150 kg, decreased by 53 kg. Her abdominal circumference was 168 cm, decreased by 31 cm. The fasting blood-glucose and postprandial blood glucose were controlled at normal levels, and no insulin or oral hypoglycemic drugs were required (Fig. 1).

After the combination of drug, behavioral and surgical therapy, various of biochemical indexes were improved in the patient. For example, the red blood cells and white blood cells were both were decreased to normal level. Besides, the creatinine and mucous strands were significantly reduced, indicating the recovery of renal function and reduction of urinary tract infection (Table 1). From the aspect of appearance, it was obvious that the patient lost weight effective, which not only be beneficial for her health, but also made her more self-confident and be willing to take part in social activities.

## 3. Results and discussion

With the development of society and the improvement of life quality, the incidence of obesity is getting higher and higher, and it has become a worldwide public health problem in recent years.^[9]^ Obesity is a chronic metabolic disease caused by multiple factors, which could cause endocrine disorders and metabolic disorders in patients.^[10]^ In this study, 2 organs of the patient have failed, and wheezing, dyspnea and other uncomfortable symptoms have seriously affected her normal life. It would be life-threatening if she was not timely treated. However, if we did the bariatric surgery immediately, it would be extremely risky because it might be difficult to remove the trachea cannula after surgery. In this dilemma, we had to make every effort to minimize the risk of the surgery.

We adopted comprehensive intervention therapy for this patient, which included diet intervention therapy, drug therapy and surgical intervention therapy.^[11]^ Diet intervention therapy means controlling patients’ daily diet and giving patients essential diet to effectively reduce the intake of saturated fatty acids.^[12]^ It was beneficial to reduce the fat content, which could not only meet the daily energy requirement and promote fat metabolism, but also promote the patient to develop healthy eating habits and change her behavior pattern to a certain extent.^[13]^ Drug treatment was mainly aimed at treating heart failure, respiratory failure and pulmonary hypertension. Some diuretic drugs were given to reduce the edema of the patient, and various measures were taken to obtain surgery opportunities, such as lowering blood sugar, reducing body weight, and reducing pulmonary hypertension. When the surgical risk was minimized, the weight of the patient had decreased to plateau, and the medicine indexes had been significantly improved, multi-disciplinary treatment would be started again to reevaluate the index of the patient. When the experts were in agreement with opinions, the bariatric surgery could be performed.

The successful treatment of this patient has a profound impact on the diagnosis and treatment of patients with extremely severe obesity and multiple complications. Firstly, multi-disciplinary combination and comprehensive treatment can effectively reduce the risk of surgery. Moreover, surgery should not be performed as soon as possible for patients with extremely severe obesity, and preoperative weight loss can improve the organs function of patients. Besides, preoperative behavioral therapy has a profound impact on the living habits of patients after surgery.^[14]^ Finally, psychological intervention can prompt them rebuild self-confidence and be willing to participate in social activities.

With the continuous development of bariatric metabolic surgery, weight loss surgery is becoming more and more mature. The patients’ various medical index can be significantly improved through the preoperative multi-disciplinary comprehensive diagnosis and treatment,^[15]^ which also provides operation with the advantages. When the time is ripe for operation, surgery and other comprehensive treatment can help patients obtain good weight loss effect and reduce complications.^[16]^

In this case, no complications occurred after the operation, the patient recovered quickly, and the blood glucose and insulin levels were decreased significantly. The patient lost weight, and she no longer needed to receive insulin and hypoglycemic drugs to control blood glucose. Therefore, from the short-term effect, the operation has a good effect on reducing weight and lowering blood glucose.^[17]^ However, the long-term therapeutic effect and the occurrence of long-term complications need to be observed persistently. At present, we need more clinical case data to verify the long-term efficacy. And we believe that combined therapy will bring new understanding and thinking to the surgical treatment of obesity and type 2 diabetes.

In conclusion, the female patient was severe obese with multiple organs failure. With the combination treatment of drug, behavioral and surgical therapy, her weight was significantly reduced, other physiological indexes were improved and the blood glucose was reduced to normal level, which promoted the overall physique to be slimmer than before and helped her rebuild confident to participate in social life.

## Author contributions

**Conceptualization:** Yuqi Gao.

**Data curation:** Qiaoni Ye.

**Formal analysis:** Qiaoni Ye.

**Software:** Jingfeng Gu.

**Supervision:** Guiqi Wang, Jingfeng Gu, liping Peng.

**Validation:** Liping Peng.

**Visualization:** Guiqi Wang.

**Writing – original draft:** Feng Feng, Jiaqi Zhang, Yuqi Gao.
